# Household cost-benefit equations and sustainable universal childhood immunisation: a randomised cluster controlled trial in south Pakistan [ISRCTN12421731]

**DOI:** 10.1186/1471-2458-5-72

**Published:** 2005-06-28

**Authors:** Neil Andersson, Anne Cockcroft, Noor Ansari, Khalid Omer, Joe Losos, Robert J Ledogar, Peter Tugwell, Beverley Shea

**Affiliations:** 1 Centro de Investigación de Enfermedades Tropicales (CIET), Universidad Autónoma de Guerrero, Acapulco, México; 2 Community Information and Epidemiological Technology (CIETcanada), Institute of Population Health, 1 Stewart Street, Ottawa, Outario, K1N GN5, Canada; 3 CIET in Pakistan, Islamabad, Pakistan; 4 Faculty of Medicine, Institute of Population Health, Ottawa, Canada; 5 CIET international, New York, USA

## Abstract

**Background:**

Household decision-makers decide about service use based largely on the costs and perceived benefits of health interventions. Very often this leads to different decisions than those imagined by health planners, resulting in under-utilisation of public services like immunisation. In the case of Lasbela district in the south of Pakistan, only one in every ten children is immunised despite free immunisation offers by government health services.

**Methods/design:**

In 32 communities representative of Lasbela district, 3344 households participated in a baseline survey on early child health. In the 18 randomly selected intervention communities, we will stimulate discussions on the household cost-benefit equation, as measured in the baseline. The reference (control) communities will also participate in the three annual follow-up surveys, feedback of the general survey results and the usual health promotion activities relating to immunisation, but without focussed discussion on the household cost-benefit equations.

**Discussion:**

This project proposes knowledge translation as a two-way communication that can be augmented by local and international evidence. We will document cultural and contextual barriers to immunisation in the context of household cost-benefit equations. The project makes this information accessible to health managers, and reciprocally, makes information on immunisation effects and side effects available to communities. We will measure the impact of this two-way knowledge translation on immunisation uptake.

## Background

Despite billions of dollars spent on childhood immunisation, some countries have never reached universal childhood immunisation (UCI), and many more have been unable to sustain it. An estimated 1.5 million deaths under 5 years of age can be prevented by vaccination each year, measles making up 37% *H. influenzae* b-related disease 30%, and pertussis 19%[1]. In many countries, there is dramatic underutilisation of the offer of free immunisation. One reason for this is a difference between understanding of the costs and benefits of vaccination in international public health circles, and what primary decision-makers for children know about immunisation and its costs.

There is a gap between the way public health specialists understand immunisation benefits, and the cost-benefit equations that household decision-makers apply to their children's immunisation. In settings like Pakistan, where an expanded program of immunisation is offered free of charge, uptake is largely determined by access to services and the attendant cost-benefit assessments by parents and caregivers. These assessments could be influenced by access to knowledge and conditioned by gender and social inequalities.

Although communication of risks and benefits by service providers can influence health-seeking behaviour [2], current approaches to health communication do not always achieve the expected results. Efforts frequently produce an increase in knowledge without a corresponding change in attitudes or behaviour[3]. This is at least partly because conventional risk communication presumes to inform an uninformed public and to reduce irrational thinking. Questioning the value of these conventional one-way knowledge transfer (KT) initiatives, more holistic perspectives take account of social and cultural influences[4]. Impact studies of communication strategies to increase vaccination in the USA, Russia and Mozambique all highlight the need of multi-channel targeting of multiple groups – families, communities, health practitioners and opinion makers, such as community and religious leaders[5-7].

Current devolution reforms in Pakistan are expected to make service delivery more effective and to support institutionalised participation of community members[8,9]. Service delivery is now a district function, yet the provinces are still responsible for planning and monitoring health services [9]. The expanded programme of immunisation (EPI) remains a federal responsibility. The 2002 Pakistan Integrated Household Survey claimed an increase in immunisation coverage, mostly in the urban areas. The proportion of fully immunised children aged 12–23 months rose from 49% to 53%, and even this small gain was not consistent countrywide. In Balochistan, where the project will be located, immunisation coverage fell from 34% in 1998–9 to 24% in 2000–1, mostly attributed to diversion of resources to the polio eradication campaigns[10].

Balochistan is Pakistan's largest province (347,000 km^2^) and has the lowest population density (6.5 million, 19/km^2^). It covers more than 40% of the land area, but has less than 5% of the national population. The disease burden in Pakistan is still largely poverty-related, much of it preventable by immunisation[11,12].

Lasbella, in the south of Balochistan is one of the province's poorest districts. In 2003 there were around 50,000 women of childbearing age in this district. There were 11,594 infants under the age of one year, and reflecting the very high infant mortality, 8,140 children aged 12–23 months. The new district administration employs 39 full time vaccinators, but still only achieves symbolic coverage. The problem does not appear to be one of supervision or management. The vaccinators report to a superintendent, who in turn reports to the Deputy District Health Officer, who reports to the Executive District Officer for Health.

## Statement of the research issue and approach

We believe that health care consumers make rational decisions from a cost-benefit perspective[13,14]. Based on their own knowledge, mothers and guardians weigh up the costs and the benefits of immunisation: how much time will it take, how much will it cost, will it work, will there be side effects and what will happen if I do not go. We propose that immunisation uptake is largely determined by this cost-benefit equation.

But the rationality of such decisions is a "bounded rationality" [15]. This concept enables health planners to see such factors as social norms and attitudes not as obstacles to rationality but rather mechanisms that facilitate fast and accurate decisions, and more efficient learning. In addition, the weigh-up of risks may be sharply discounted between the time of the immunization and the time of possible infection. Also, gender and poverty probably affect the household cost-benefit equation. The poor, who typically have less access to services and less information about services, almost certainly weigh up the costs and benefits in a different way than do the rich [16]. Diseases like measles and pertussis may be an inconvenience for the well-nourished, whereas for the malnourished, they can be a question of life or death. Costs of *not *vaccinating (disease burden, care and funerals) are borne disproportionately by the poor; in a single epidemic, these diseases can destroy a household economy[17].

### The household cost-benefit equation is, therefore, a lens through which to view immunisation and the obstacles to immunisation

A focus on these equations gives value to the way ordinary people see immunisation, allowing their views to be taken systematically into account. It gives primacy to household decisions, and recognises that communication about immunisation is a two-way street. We propose to test the importance of this household cost-benefit equation that decision-makers for children derive from their knowledge, attitudes, social norms, intentions, sense of agency and degree of socialisation about immunisation.

The hypothesis is that this dynamic equation can be influenced by two-way knowledge translation (KT), and based on this culture-appropriate exchange, that people will adjust their household cost-benefit equations and their uptake of immunisation. A corollary of the household cost-benefit equation is accessible to planners and health service managers: cost-gains. Derived from the same data used by communities for their cost-benefit equations, cost-gains offers a common language for interaction between health services and communities. If local health managers understand how primary decision makers assess immunisation – their cost-benefit equations – this can improve their engagement with communities and lead, in turn, to sustainable UCI.

## Methods

### Objective 1. Identify the *barriers and information imbalances *that reduce childhood immunisation, in particular increasing understanding of the household cost-benefit equations underlying uptake of immunisation

Randomised controlled cluster trials are fairly widely used in developed countries, and have been introduced in several developing countries[18-20].

#### Randomisation

From the latest Pakistan census, 32 enumeration areas were randomly selected to represent the population of Lasbela district. Once the baseline survey was completed, clusters were randomised into intervention and control, with precautions about the usual biases of this design[21]. In each cluster, interviewers contacted homes of 100 children under the age of 24 months of age (a total of 3344 households). Three successive cycles that examine successive cohorts of children in this age group, and the same number of households in each site (not necessarily the same households) will preserve the proportional representation.

#### Design of survey instruments

Questionnaires were adapted from international EPI standards. Additional fact-finding tools will produce qualitative evidence – key informant interviews, service worker questionnaires, protocols for institutional reviews and focus group discussions. The results of each round might identify additional stakeholder-driven issues and priorities and be used to refocus the subsequent survey cycles.

#### Survey content

In addition to baseline data about the coverage with and obstacles to immunisation, we enriched the standard KAP approach[22] with a behaviour change model adapted by CIET to measure youth responses to risk. The beyond-KAP approach, "cascada", refers to **c**onscious knowledge about immunisation and its side effects, **a**ttitudes to childhood immunisation, **s**ocial norms (what neighbours do) and positive or negative deviation from those norms, intentions to **c**hange or to vaccinate in the future, **a**gency (expectancy of self-efficacy or collective efficacy) and **d**iscussion about immunisation, its benefits and side effects. The outcome of this "cascada" is the **a**ction, immunisation. We will document perceived and real costs of immunisation and non-immunisation, and the household weigh up of costs and benefits.

#### Piloting

Eight rounds of piloting in non-sample sites included testing new sections of the instrument, testing the instrument for flow, and then testing the instrument as a whole in order to finalise the process. The pilot exercises assisted in refining the instruments, testing for clarity and ensuring proper translation.

#### Ethical review

Two review panels, one at the University of Ottawa and a panel in the south of Pakistan registered with the US Government's Office of Human Research Protections, deliberated the ethical issues and approved the study.

### Objective 2. Formulate and implement knowledge transfer based on household cost-benefit equations, compared with health information in reference (control) communities

#### Intervention

We will update and translate available knowledge on immunisation and combine this with data from the baseline as an intervention focussed on the household cost-benefit equation. Mass media channels can increase awareness and knowledge, but interpersonal channels seem to work better in changing attitudes and behaviour[23,24]. A combination of communication channels could, therefore, include mass media appeals, reminder systems and engagement through trusted sources, and addressing risks and benefits of vaccination in an understandable manner[25-28]. Upon consideration of the evidence, people will hopefully adjust their household cost-benefit equations. We will measure this adjustment in the subsequent cycles.

#### Analysis

We will estimate the impact of this knowledge transfer on changing beliefs and practices of decision-makers for children and, as a consequence, immunisation uptake. Differences between intervention and reference sites will be analysed for independence from age, sex, household employment, community size, remoteness and other factors. Risk analysis will rely on the Mantel-Haenszel procedure and contrasts reported as the odds ratio (OR) or risk difference (RD)[29-31]. In the analysis, each site or cluster is treated as a mini-universe characterised by certain social dynamics, history, culture and collective practices. Because qualitative data are coterminous (they coincide with the same population) with the individual questionnaires it is easy to link quantitative and qualitative data[32]. An unconditional logistic regression model will be developed where appropriate, using a step-down approach from a saturated model[33].

We also seek to express a planning-appropriate perspective that fits the household cost-benefit perspective. 'Gain' is the theoretical proportion of the *entire *population that stands to benefit from the removal of an obstacle or the universalisation of an intervention. It is calculated by multiplying the risk difference (risk among exposed minus risk among unexposed) by the proportion requiring intervention (those exposed, if the interest is to remove risk factors).

### Objective 3. Measure the impact of the KT on coverage and attitudes about immunisation

#### Focus groups to identify strategies

After the preliminary analysis, field teams will return to the communities to hold gender stratified focus groups. Key findings from the household interviews will be shared with the groups to generate additional insights, including how best to let similar communities know about the findings. Focus groups typically involve 8–12 participants. In a quiet location, groups are limited to a maximum of one hour in recognition of the value of participants' time. Participants are reassured about confidentiality (no identifiers are recorded). A trained facilitator runs the group, prompts to provoke discussion, and encourages participants to express opinions. A second member of the team records the content and manages the time.

##### Communications

Communication strategies (see below) to share the outcomes of the measurement with stakeholders in the intervention communities will be heavily conditioned by the outcome of the focus groups. The core concept is to socialise the household cost-benefit equation. Strategies are likely to include work with local elected representatives, community and religious leaders, service workers and community action groups such as citizen community boards (CCBs)[34].

###### Repeat survey

Each year for three years, the measurement will be repeated in both intervention and control sites. Much of the key instrument content will be unchanged, to detect time trends. Responses will provide substrate for the next round of household cost-benefit equations and cost-gain analysis, which in turn will feed into the next round of intervention.

### Objective 4. Develop an *evidence-based and gendered systems approach to increasing equity in immunisation*, rooted in community knowledge, capable of building on local health protection cultures and of informing evidence-based decision-making to improve the health of populations and strengthen health systems through immunisation services

A lot of effort has gone into the supply side of immunisation (vaccine purchase, training health workers and logistics). There is a need to focus systematically on the *demand *for immunisation – which is probably a direct function of the quality of information people have.

Parallel to the community-based knowledge transfer intervention, the team will work with the district authorities in Lasbela. We will build capacity to improve immunisation rates in the selected district, reaching health care workers, community leaders and policy makers. Research teams will be trained in community-based research, enhancing the capacity for ongoing monitoring of immunisation and other key public services.

The cost-gains approach offers a bridge between planner and community views. Proving the value of this parameter could support a paradigm shift in resource allocation, from a system based on reconciliation of competing sectoral claims without a comparable evidence base to cost-gain planning.

The main selling point of a new knowledge transfer approach to sustainable immunisation is that it must work. immunisation coverage must increase measurably and people in key positions must know about this. Mainstreaming the household cost-benefit equation begins with respectful dialogue with local health and political authorities about immunisation concepts, service delivery, effects and side effects in the communities. The central activity is then to demonstrate by measuring and communicating, in reiterative cycles, the effect on cost and benefit assessments. Comparisons between sites with different levels or types of intervention will be almost as informative as the longitudinal picture emerging by following each site over four years. Evaluation is thus built into the project.

In addition to the overall concept of evidence-based immunisation support, and the household cost-benefit equation, relevant procedures and tools include:

1. Protocols for the survey (household beyond-KAP, key informants and institutional review) and sample selection, processing results through double data entry, epidemiological analysis and interpretation in community-based focus groups;

2. Procedures for achieving policy level buy-in, including aggregation tools (like customised epidemiological mapping freeware) that allow compounding of local experiences into regional and national pictures; a menu of methods and practical examples of communication tools for opening evidence-based dialogue that can increase community ownership of immunisation.

## Discussion

Engagement of communities in evidence-based planning is a novel approach to immunisation, an intervention driven perhaps more than any other by an inappropriate if well-intentioned ethos of "we know what is good for you". The idea of a household cost-benefit equation is common sense: people weigh things up before their health choices. This study hopes to answer questions about what it takes to enter a dialogue that influences these equations. The year-to-year shift in knowledge, attitudes, subjective norms, intention, sense of agency, ability to discuss, and ultimately, uptake of immunisation will be evident from the results of the successive cycles.

Best practice cases – communities that increase immunisation – will be identified and held up as positive examples of what is possible under prevailing conditions. The direct beneficiaries will be the public, but indirect beneficiaries will be planners and policy-makers in federal, provincial and district governments. Improved understanding of immunisation risks and cost implications of higher immunisation uptake may be applicable in many other countries. Proof of cost-gains as the planners' corollary of the household cost-benefit equation could open new horizons for evidence-based service-public interaction.

## Competing interests

The author(s) declare that they have no competing interests.

## Contributions of authors

NA designed the study, developed the methodology and wrote the proposal. NA designed CIET map freeware, included in the proposal to combine mapping and epidemiology for decision makers. He is responsible for overseeing the project.

AC reviewed the protocol and is responsible for overseeing the project in Pakistan.

NoorA conducted the pilot studies, contributed to the instrument design and supervises the fieldwork in Balochistan.

KS coordinates the project in Pakistan.

JL reviewed the protocol and contributed to the overall study plan.

RJL co-developed the concept of household cost-benefit equations, and contributed to the proposal and development of instruments.

PT reviewed the protocol and will contribute to the systematic review.

BS helped write the proposal and is responsible for the systematic review.

## Pre-publication history

The pre-publication history for this paper can be accessed here:


